# Full-information item bifactor model for mathematical ability assessment in Chinese compulsory education quality monitoring

**DOI:** 10.3389/fpsyg.2022.1049472

**Published:** 2022-12-12

**Authors:** Xiangbin Meng, Tao Yang, Ningzhong Shi, Tao Xin

**Affiliations:** ^1^School of Mathematics and Statistics, KLAS, Northeast Normal University, Changchun, China; ^2^Collaborative Innovation Center of Assessment for Basic Education Quality, Beijing Normal University, Beijing, China

**Keywords:** full-information bifactor item factor model, mathematical ability, item response theory, China compulsory education quality monitoring, large scale testing

## Abstract

This study focuses on the measurement of mathematical ability in the Chinese Compulsory Education Qualification Monitoring (CCEQM) framework using bifactor theory. First, we propose a full-information item bifactor (FIBF) model for the measurement of mathematical ability. Second, the performance of the FIBF model is empirically studied using a data set from three representative provinces were selected from CCEQM 2015–2017. Finally, Monte Carlo simulations are conducted to demonstrate the accuracy of the model evaluation indices and parameter estimation methods used in the empirical study. The obtained results are as follows: (1) The results for the four used model selection indices (AIC, SABIC, HQ, BIC) consistently showed that the fit of the FIBF model is better than that of the UIRT; (2) All of the estimated general and domain-specific abilities of the FIBF model have reasonable interpretations; (3) The model evaluation indices and parameter estimation methods exhibit excellent accuracy, indicating that the application of the FIBF model is technically feasible in large-scale testing projects.

## 1. Introduction

The Chinese Compulsory Education Qualification Monitoring (CCEQM) project (The National Assessment Center for Education Quality, 2018), which is organized by the Basic Education Quality Monitoring Centre (BEQMC) under the Ministry of Education of the People's Republic of China (MOE), is the largest student assessment project in China. CCEQM applies to mathematics, Chinese reading, science, moral education, art, and physical education. Each subject is monitored every 3 years, with a focus on two subjects per year (Jiang et al., 2019). The first assessment cycle ran from 2015 to 2017, with a total of 572,314 fourth-grade and eighth-grade students from 32 Chinese provinces, municipalities, and autonomous regions participating in the assessment (Yin, 2021). In July 2018, the first CCEQM Report was released, attracting considerable attention in China. Furthermore, CCEQM 2015–2017 evaluated students' academic achievements based on the concept of core literacy. Therefore, the work conducted toward CCEQM 2015–2017 provides valuable experience for educational evaluation reform in China.

Mathematics is one of the most important basic subjects in the compulsory education stage of China, and mathematical literacy is the core content assessed by CCEQM 2015–2017. On the basis of the “10 core concepts” put forward by the “Mathematics Curriculum Standard of Compulsory Education (2011 Edition),” and referring to the experience of international large-scale assessment projects such as the Programme for International Student Assessment (PISA) or the Trends in Mathematics and Science Study (TIMSS), a mathematics literacy assessment framework was developed by the experts of BEQMC for CCEQM 2015–2017. Specifically, in the mathematics literacy framework, mathematical ability—as a general concept—includes the five domains of “mathematical computation,” “space imagination,” “data analysis,” “logical reasoning,” and “problem solving” (The National Assessment Center for Education Quality, 2018; Jiang et al., 2019). The first four are the same as those in the mathematics literacy framework developed by the “Mathematics Curriculum Standards of Senior High School (2017 Edition)” and the “Mathematics Curriculum Standards of Compulsory Education (2022 Edition).” The domain of “problem solving” was defined by reference to PISA 2012 (OECD, 2014), and covers the ability to discover, analyze, and solve problems. The definitions of mathematical ability, as well as the five domains, are not the focus of this study, so they are not described in detail here.

In CCEQM 2015–2017, the subscores on the five cognitive domains for mathematics are estimated using between-item multidimensional IRT (MIRT) models, and the unidimensional IRT (UIRT) model is used to estimate the overall score for mathematics (Jiang et al., 2019). Note that the UIRT model is also the main measurement model in PISA (OECD, 2014). There are some issues that need attention. First, the subscores on the five domains must be strongly correlated, because all five domains share common cognitive and intelligence influences. The common element is not captured in the between-item MIRT models, which is likely to result in a false interpretation of the subscores. However, the one-factor structure of the UIRT model does not match the five-dimensional assessment framework, in which only the common element is considered, and so the idiosyncratic nature of each domain cannot be explained. Furthermore, as discussed by Jiang et al. (2019), the subscores are hardly comparable with the overall score, as they are obtained from different models. Therefore, it is desirable to develop a more powerful and reasonable measurement model for the evaluation of mathematical ability in large-scale assessment projects.

Bifactor models are a powerful approach for representing a general construct comprised of several highly correlated domains (Chen et al., 2006; Bornovalova et al., 2020), in which the common and unique elements of all domains are modeled separately. The bifactor theory and model were originally proposed by Holzinger and Swineford (1937) to rectify the problem of adequately separating a single general factor of intelligence (Spearman, 1904) from domain factors. To analyze item response data using a bifactor structure, Gibbons and Hedeker (1992) and Gibbons et al. (2007) generalized the work of Holzinger and Swineford (1937) to derive full-information item bifactor (FIBF) models for dichotomous and polytomous response data, respectively. Cai et al. (2011) further extended the FIBF framework to a multiple-group model that supports a variety of MIRT models for an arbitrary mixture of dichotomous, ordinal, and nominal items. After years of relative neglect, bifactor analysis has become an important statistical method for handling multidimensional concepts. The bifactor model has been used primarily in studying intelligence and personality (Gault, 1954; Acton and Schroeder, 2001; Rushton and Irwing, 2009a,b; Watkins, 2010; Martel et al., 2011; McAbee et al., 2014; Watkins and Beaujean, 2014; Cucina and Byle, 2017; Moshagen et al., 2018). Recently, it has become increasingly popular across a broad range of research fields such as depression and anxiety (Simms et al., 2008; Gomez and McLaren, 2015; Kim and Eaton, 2015; Olatunji et al., 2017; Snyder et al., 2017; Jorge-Botana et al., 2019; Heinrich et al., 2020; Waldman et al., 2020; Arens et al., 2021; Caiado et al., 2022), health outcomes (Reise et al., 2007; Leue and Beauducel, 2011; Shevlin et al., 2016; Monteiro et al., 2021), emotion expression (Caiado et al., 2022), and cognitive abilities (McFarland, 2013, 2016; Beaujean et al., 2014; Valerius and Sparfeldt, 2014; Foorman et al., 2015). In addition, as a special case of confirmatory MIRT modeling, FIBF models have been used to address some important problems in psychological and educational measurement. For instance, modeling test response data and identifying the local dependence of item responses (DeMars, 2006; Liu and Thissen, 2012), assessing the dimension of test scales (Immekus and Imbrie, 2008), and equating and vertical scaling of test scores (Li and Lissitz, 2012; Kim and Cho, 2020). It is apparent that the advantages and values of bifactor analysis have been largely verified and are widely recognized.

Motivated by previous studies, this article proposes a bifactor structure for mathematical ability, and applies a mixed FIBF model to assess mathematical achievement. Furthermore, an empirical study is conducted based on data from three representative provinces sampled for the CCEQM 2015–2017 survey. The main tasks of this empirical study are to verify the advantages of the FIBF model over the traditional models of between-item MIRT and UIRT, and to interpret the bifactor scores of mathematical ability. Furthermore, to ensure the accuracy of the empirical analysis, a Monte Carlo simulation study is conducted to investigate the performance of the statistical analysis methods used in the empirical study. Finally, we summarize the research conclusions and review the main results of this study, while identifying its limitations, and discuss some future research issues.

## 2. FIBF model for mathematical ability in CCEQM 2015–2017

The Mathematics Curriculum Standards of Senior High School (2017 Edition) stated that “...different domains of mathematical literacy are not absolutely different, but integrated with each other...” So the five domains (“mathematical computation,” “space imagination,” “data analysis,” “logical reasoning,” and “problem solving”) are different and represent different aspects of mathematical ability, but they must have a overlap or common part. From this point of view, we consider that the bifactor structure is suitable for modeling the assessment of mathematical ability.

**Figure 1:**
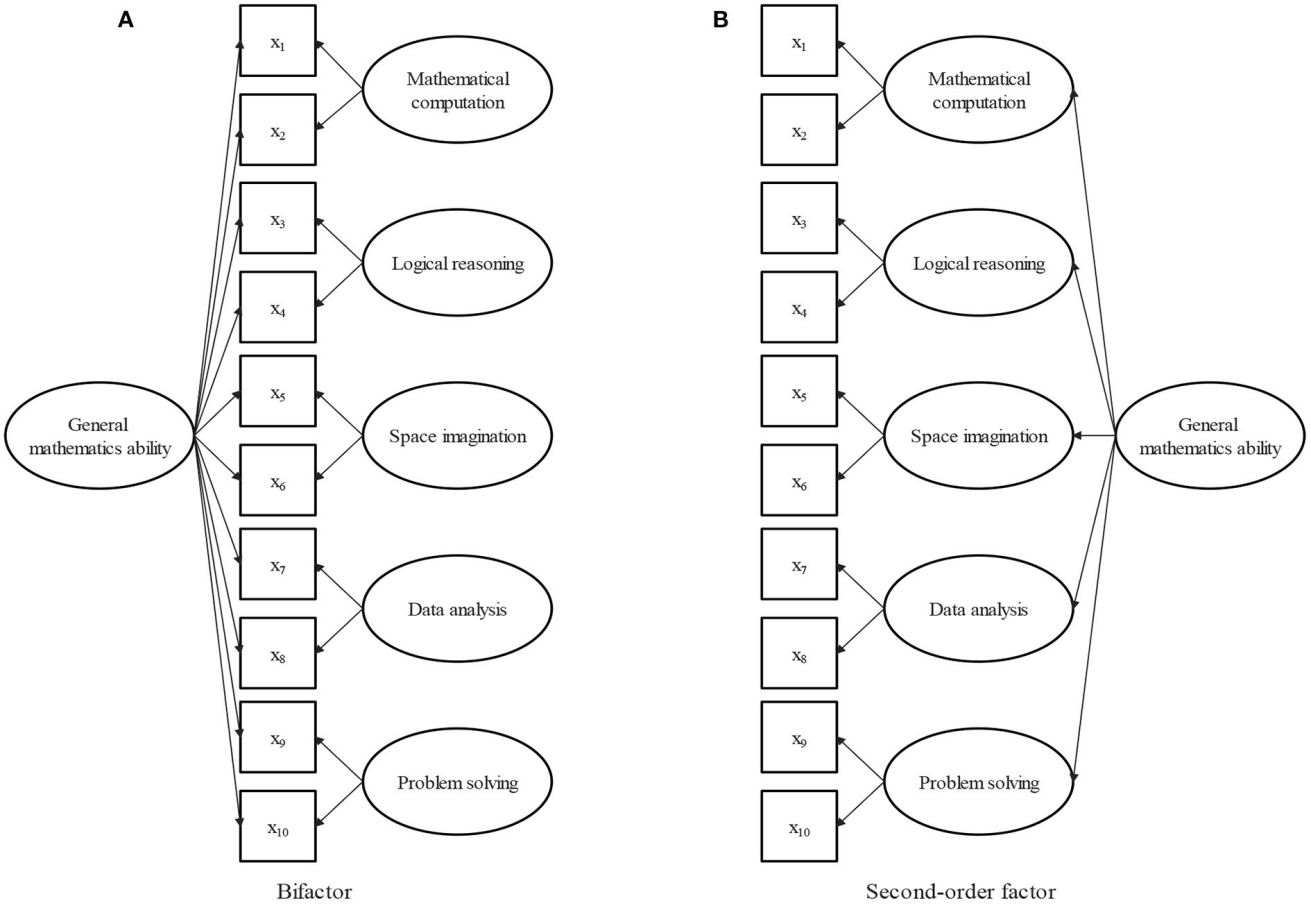
**(A)** Bifactor structure of mathematical ability. **(B)** Second-order factor structure of mathematical ability.

The item bifactor measurement structure for mathematical ability is illustrated in Figure 1A, where the observed categorical responses are indicated by squares, the latent factors are represented by circles. All items load on a general or common factor, although each item loads on only one group or domain-specific factor. The general factor, which represents the common element of all aspects of mathematical ability, is interpreted as the general mathematical ability, which is a broadly defined concept. The five group factors represent the unique elements of the five domains, and can be interpreted as domain-specific mathematical abilities that are conceptually more narrowly defined mathematical facets. The particularity and commonality of mathematical ability are represented together in the bifactor structure.

When there is no group factor, the bifactor structure is reduced to a one-factor structure; when there is no general factor, it is a five-factor structure. From this point of view, the bifactor model can be thought of as a combination of one-factor and five-factor structures. Because the commonality of the five domains of mathematical ability is explained by the general factor, the general and group factors are assumed to be orthogonal in the bifactor analysis. The orthogonality of general and specific factors is beneficial for evaluating the relative contribution of each factor to the overall test performance. In the following, an FIBF model is proposed for the math test item response data under the bifactor structure of mathematical ability.

Consider *j* = 1, ..., *M* items in the total item pool, each scored in *K*_*j*_ ≥ 2 categories, where *K*_*j*_ = 2 indicates that the item is scored dichotomously. Let there be *i* = 1, ..., *N* independent students, and let *X*_*ij*_ (with *x*_*ij*_ representing one observation) denote the response variable from person *i* to item *j*. Without loss of generality, we assume that *X*_*ij*_ takes integer values from {0, 1, ..., *K*_*j*_ − 1}. Let θ_*i*_ = (θ_0*i*_, θ_1*i*_..., θ_5*i*_) denote the vector of the latent mathematical ability of student *i*, wherein θ_0*i*_ denotes the general mathematical ability and (θ_1*i*_..., θ_5*i*_) denote the five domain-specific mathematical abilities. The mathematics testing instrument consists of two types of items: dichotomously scored items and graded scored items. Thus, the FIBF model must be a mixed dichotomous and polytomous model. To represent the dichotomous item response data, the bifactor extension of the 2-parameter logistic (2PL) model is used. The 2PL is one of the most important dichotomous IRT models, and is widely used in practice. When item *j* is a dichotomous item, *X*_*ij*_ ∈ {0, 1}; *X*_*ij*_ = 1 denotes the correct response, and *X*_*ij*_ = 0 otherwise. The conditional probability of the correct response given θ_0*i*_ and θ_*vi*_ is formulated as


(1) $$\begin{array}{l} P\left(X_{ij}=1|\theta_{0i},\theta_{vi}\right)=\frac{\exp\left(a_{0j}\theta_{0i}+a_{vj}\theta_{vi}+b_{j}\right)}{1+\exp\left(a_{0j}\theta_{0i}+a_{vj}\theta_{vi}+b_{j}\right)}, \end{array}$$

where *a*_0*j*_ and *a*_*vj*_ are the item slopes, which are analogous to the factor loading parameters on the general factor and the domain-specific factor, and *b*_*j*_ is the item intercept parameter.

The bifactor extension of the logistic version of GRM is used to represent the graded item response data. When item *j* is graded, *X*_*ij*_ ∈ {0, 1, ..., *K*_*j*_ − 1} and *K*_*j*_ > 2. The conditional probability of response category *k* given θ_0*i*_ and θ_*vi*_ is formulated as


(2) $$\begin{array}{l} P(X_{ij}=k|\theta_{0i},\theta_{vi})=P(X_{ij}\geq k|\theta_{0i},\theta_{vi})-P(X_{ij}\geq k\;\\ \;+1|\theta_{0i},\theta_{vi}), \end{array}$$

and


(3) $$\begin{array}{lll} P\left(X_{ij}\geq 0|\theta_{0i},\theta_{vi}\right) & = & 1, \end{array}$$

(4) $$\begin{array}{l} P(X_{ij}\geq k|\theta_{0i},\theta_{vi})=\frac{\exp(a_{0j}\theta_{0i}+a_{vj}\theta_{vi}+b_{kj})}{1+\exp(a_{0j}\theta_{0i}+a_{vj}\theta_{vi}+b_{kj})},\;\\ \;k=1,..,K_{j}-1, \end{array}$$

(5) $$\begin{array}{lll} P\left(X_{ij}\geq K_{j}|\theta_{0i},\theta_{vi}\right) & = & 0, \end{array}$$

where *b*_1*j*_, ..., *b*_(_*K*__*j*_ − 1)*j*_ are the set of *K*_*j*_ − 1 (strictly ordered) intercepts. As before, *a*_0*j*_ and *a*_*vj*_ are the item slope parameters.

Importantly, the statistical inference (such as parameter estimation and model fit evaluation) using the FIBF models is mature, and a number of software packages have been developed. At present, the application of the FIBF model is technically feasible in modeling the incomplete mixed item response data that often occur in large-scale testing projects. Zhan et al. (2019) proposed a third-order DINA model, which is a cognitive diagnosis model, for assessing scientific literacy in PISA 2015. As verified by Zhan et al. (2019), this third-order Deterministic Inputs Noisy “And” gate model (DINA) model has some advantages over the UIRT model for modeling scientific test data. However, the DINA model is dichotomous, and cannot model polytomous response data, which greatly limits its application in large-scale assessment projects. From this point of view, practicality and feasibility are important benefits of using the FIBF model to measure mathematical ability.

The second-order factor model is an alternative method for representing general constructs consisting of multiple highly related domains (Chen et al., 2006). The second-order factor structure for mathematical ability is illustrated in Figure 1B, in which the five domains of mathematical ability are explained by defining five first-order factors, and correlations among the five domains are identified by stipulating a single second-order factor. In the second-order model, general mathematical ability is conceptualized in terms of a second-order factor. Different from the bifactor model, the effects of the second-order factor on the item responses are mediated by the five first-order factors. Consequently, the first-order factors reflect two sources of variance (general and group), while the group factor in the bifactor model only reflects group effects. The bifactor model directly separates the unique contributions to the item responses of the general and group factors. Compared with the second-order model, the bifactor model makes evaluating theoretical hypotheses about general and group factors clearer and more interpretable. Furthermore, the second-order model is simply a more constrained version of the bifactor model. The second-order structure can be derived from the bifactor structure by constraining the ratio of the weights between any given specific factors and keeping the general factor constant (Reise, 2012). Overall, in contrast to the second-order model, the bifactor model makes theoretical hypotheses more interpretable, and has more degrees of freedom with which to fit the data. In addition, several studies have verified that bifactor models can produce a better fit than second-order models (Morgan et al., 2015; Cucina and Byle, 2017; Bornovalova et al., 2020).

## 3. Empirical study

### 3.1. Data description

Data from three representative provinces were selected from the CCEQM 2015–2017 survey, in which 2,017 fourth-grade students (53% males) and 1,404 eighth-grade students (54% males) participated. To ensure the representativeness of the data, three provinces were selected from different geographical regions (east, middle, and west), economic development levels (developed, moderately developed, and less developed), and mathematical academic achievement ranking (high, middle, and low).

Let us introduce the design of the mathematical assessment instrument in CCEQM. To ensure broad content coverage while avoiding an excessive testing burden, the partial balanced incomplete block design was employed to administer the math tests. Specifically, for the fourth-grade students, 59 items were allocated to six test booklets, each booklet consisting of 10 dichotomous items and 8 polytomous items; for the eighth-grade students, a total of 60 items were grouped into six test booklets, each booklet consisting of 12 dichotomous items and 8 polytomous items. Each student was assessed with only one booklet and each booklet was completed by several of the students. In this way, the testing time was held to <2 h, and all five domains of mathematical literacy could be adequately covered. However, the incomplete test administration design resulted in incomplete test data, which increases the difficulty of data analysis. In this empirical study, the R package “mirt” was used to conduct the statistical analysis. This package allows for statistical inferences on multidimensional item response models under incomplete test administration; additionally, it is open source and can be easily obtained.

Three competing models were fitted to the data, namely the FIBF (bifactor structure), between-item MIRT (correlated-factor structure), and UIRT (one-factor structure) models. The MIRT was estimated using the Metropolis-Hastings Robbins-Monro (MH-RM) algorithm of Cai (2010), whereas the FIBF and UIRT were estimated using the expectation-maximization algorithm, with the mathematical abilities of students estimated using the expectation *a posteriori* (EAP) estimation. These estimation methods are commonly used in practice and are known to be powerful. Bifactor models are more general than one-factor, correlated-factor, and second-order factor models, and are thus more prone to overfitting (DeMars, 2006; Murray and Johnson, 2013; Rodriguez et al., 2016; Bonifay and Cai, 2017; Greene et al., 2019; Sellbom and Tellegen, 2019), that is, a bifactor model is inappropriately favored by model selection indices. Therefore, overfitting is an important issue in the use of bifactor models. To avoid unreasonable model fitting, the four commonly used model selection indices of Akaike's information criterion (AIC; Akaike, 1987), Bayesian information criterion (BIC or Schwarz criterion; Schwarz, 1978), Hanna–Quinn index (HQ; Hannan and Quinn, 1979), and sample size-adjusted BIC (SABIC; Sclove, 1987) were computed to compare the model fitting.

### 3.2. Results

#### 3.2.1. Comparison of models

First, the obtained values of the four model selection indices (AIC, SABIC, HQ and BIC) for the three competing models are reported in Table 1. The four model selection indices of the FIBF model are consistently smaller than those of the other two models, with the largest values given by the between-item MIRT model. These results consistently support the FIBF model as the best for fitting this empirical data. The fit of the UIRT model is better than that of the MIRT model.

**Table 1:** Four model selection indices (AIC, SABIC, HQ, and BIC) of the three competing models (FIBF, MIRT, and UIRT) for fitting the mathematics test data of the fourth and eighth grades in CCEQM 2015–2017.

		**AIC**	**SABIC**	**HQ**	**BIC**
Fourth grade	FIBF	**45916**	**46422**	**46342**	**47029**
	MIRT	46199	46586	46525	47088
	UIRT	46166	46528	46471	47068
Eighth grade	FIBF	**29213**	**29640**	**29617**	**30294**
	MIRT	30363	30684	30667	31176
	UIRT	29613	29916	29802	30380

The smallest values of the four model selection indices are bold.

**Figure 2:**
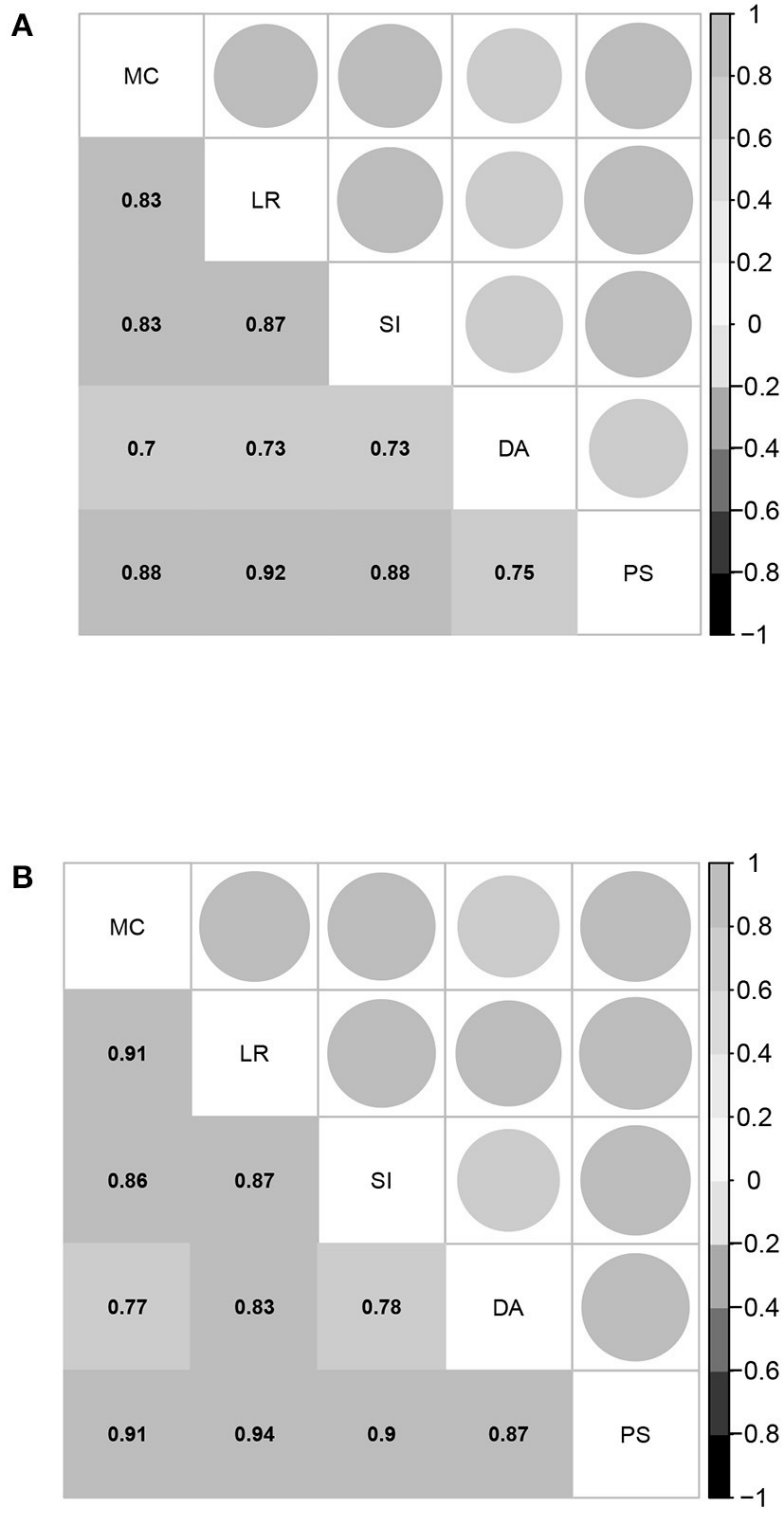
Estimated correlation coefficients between the five domain-specific abilities of the between-item MIRT model for students in the fourth and eighth grades. MC, mathematical computation; LR, logical reasoning; SI, space imagination; DA, data analysis; PS, problem solving. **(A)** Fourth grade. **(B)** Eighth grade.

Further, the estimated correlation coefficients of the five latent abilities of the between-item MIRT model are given in Figure 2. All values are larger than 0.7, and most of them are above 0.8, indicating that the five domains of mathematical ability are highly related. The strong correlations among the five domains of mathematical ability once again support the assertion that the bifactor structure is suitable for representing mathematical ability. Based on these results, it is not surprising that the four model selection indices consistently demonstrate that UIRT is superior to between-item MIRT, because a between-item MIRT model with a high related factor structure is close to the UIRT model, and the model evaluation indices prefer simpler models.

#### 3.2.2. Correlation analysis of the factor scores

**Figure 3:**
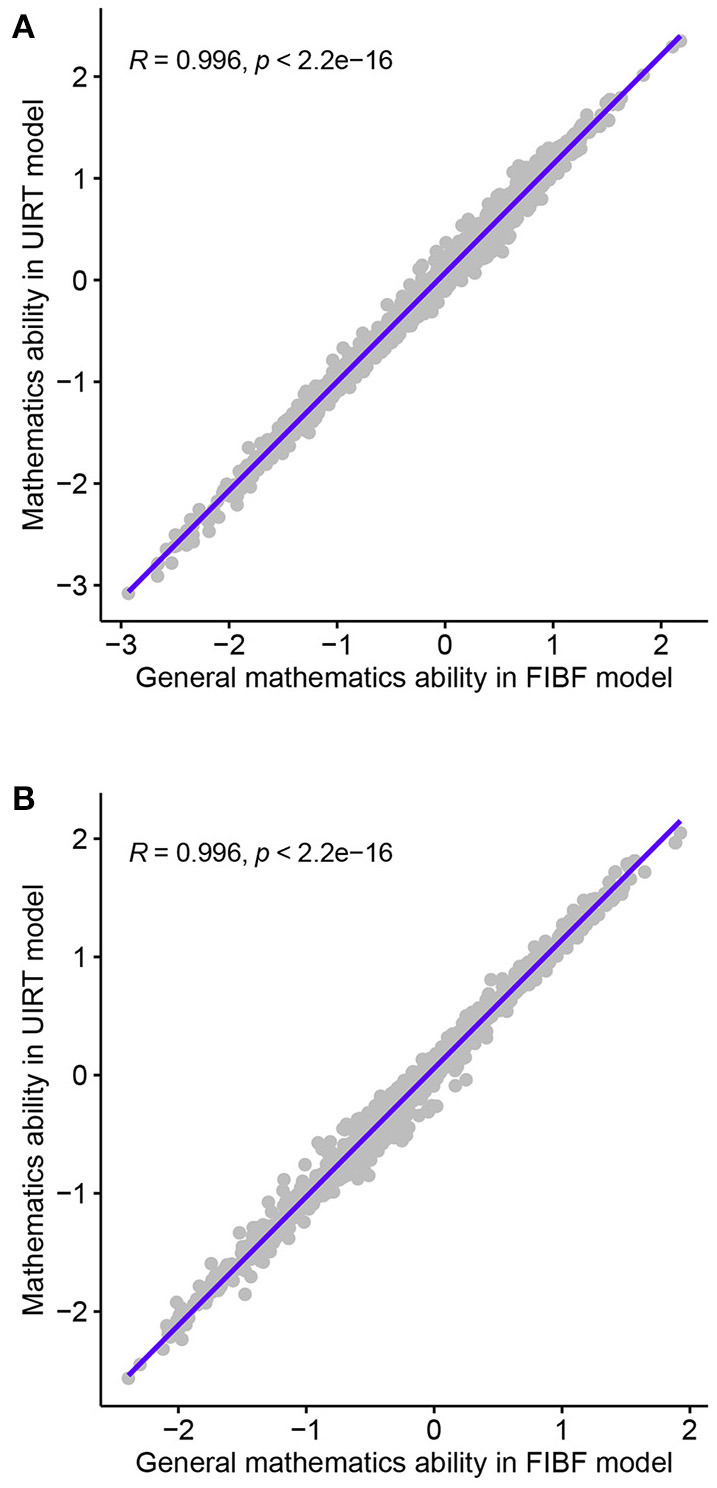
Correlation coefficients between the general abilities of the FIBF model and the abilities of the UIRT model for students in the fourth and the eighth grades. **(A)** Fourth grade. **(B)** Eighth grade.

To investigate the performance of the FIBF model, the relationships between the mathematical ability scores from the FIBF model and those from the between-item MIRT and UIRT models were analyzed. First, the correlation coefficients between the general ability of the FIBF and the ability parameter of the UIRT model (denoted as *R*) were computed; the results are shown in Figure 3. Almost all points fall on the diagonal, and the correlation coefficients for both the fourth- and eighth-grade students are *R*≅0.996, that is, they are approximately equal to 1.00. This indicates that the general mathematical ability reflected by the FIBF model is nearly equal to the ability suggested by the UIRT model. This phenomenon supports the assertion that general factor of the FIBF model can be interpreted as the general mathematical ability and represents the common element of the five domains of mathematical ability.

**Figure 4:**
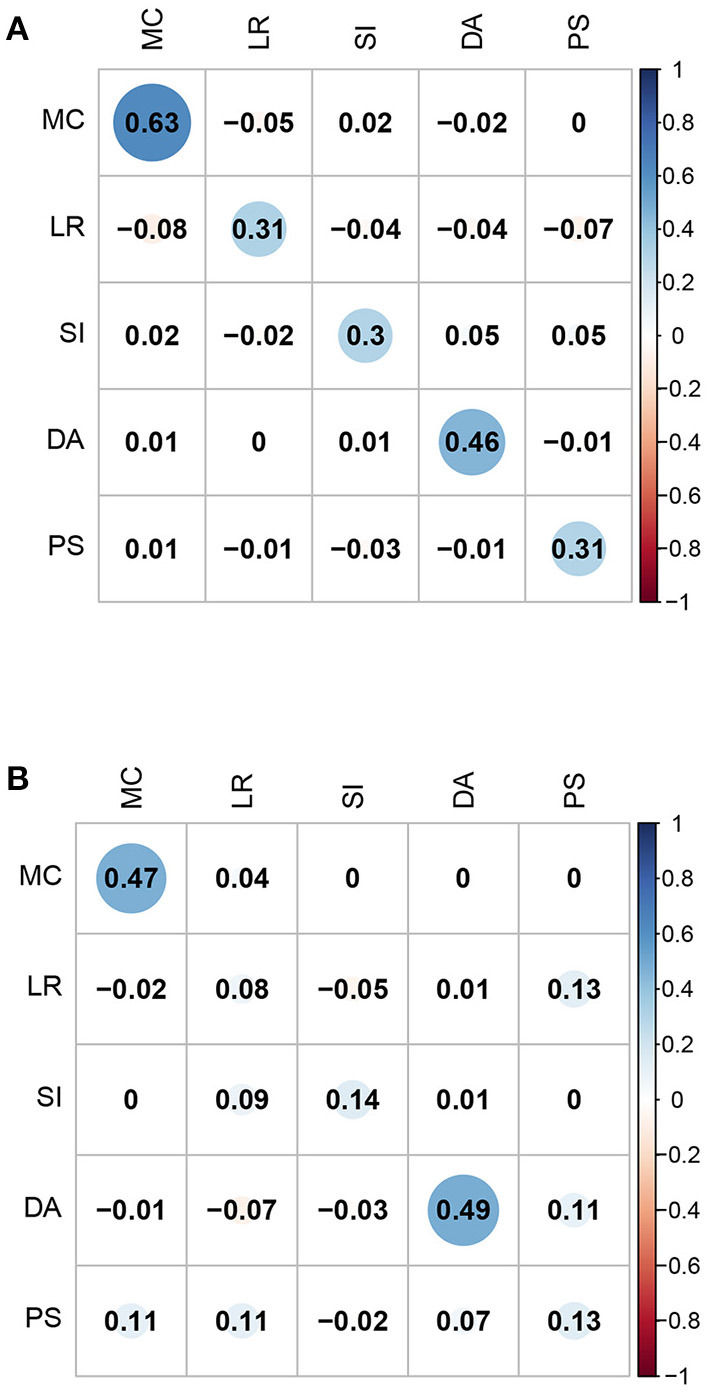
Correlation coefficients between the five domain-specific abilities of the FIBF model and those of the between-item MIRT model. MC, mathematical computation; LR, logical reasoning; SI, space imagination; DA, data analysis; PS, problem solving. **(A)** Fourth grade. **(B)** Eighth grade.

**Figure 5:**
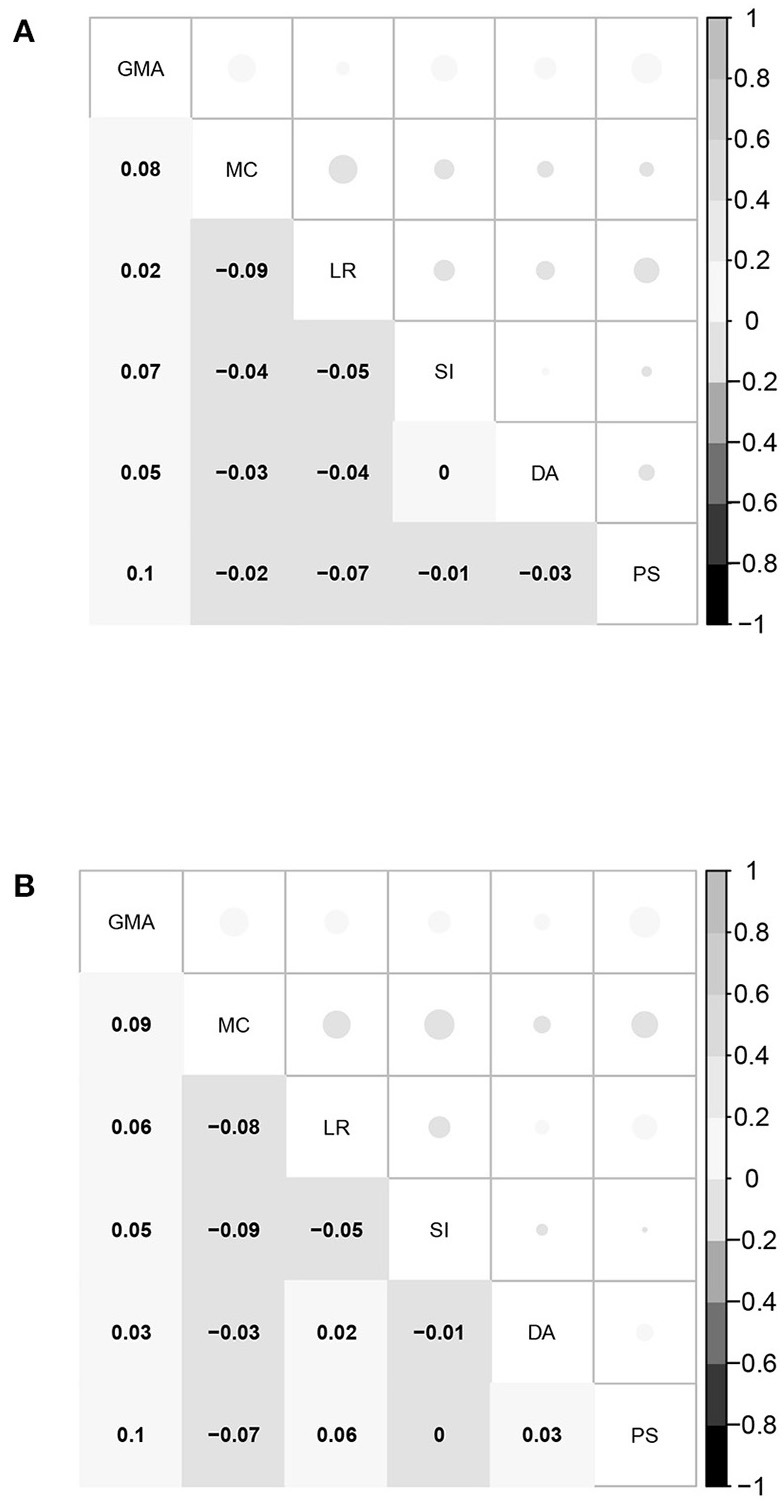
Correlation coefficients between the six abilities (the general ability and the five domain-specific abilities) of the FIBF model for students in the fourth and eighth grades. GMA, general mathematical ability; MC, mathematical computation; LR, logical reasoning; SI, space imagination; DA, data analysis; PS, problem solving. **(A)** Fourth grade. **(B)** Eighth grade.

Second, the correlation coefficients between the five domain-specific mathematical abilities of the FIBF model and those of the MIRT model were computed; the results are presented in Figure 4. For the fourth-grade students, the correlation coefficients of the same domain-specific mathematical ability from the two models are between 0.3 and 0.6, and exhibit moderate positive correlations; for the eighth-grade students, these correlation coefficients are slightly smaller. However, for both grades, the correlation coefficients between different domain-specific mathematical abilities from the two models are close to 0.0. Based on these results, it can be concluded that the group factors of the FIBF model represent the unique elements of the five specific domains. Additionally, we conducted a correlation analysis of the estimated latent factors in the FIBF model, which reflects whether the latent factors are orthogonal; the results are presented in Figure 5. All of the correlation coefficients are very close to 0.0, which strongly indicates that the general and domain-specific latent abilities are independent. In addition, due to the independence of general and specific factors, the relative contribution of each to the overall test performance can be evaluated more readily.

Overall, the results obtained in this empirical study demonstrate that the bifactor model is a powerful approach for representing the intercorrelations among the five domains of mathematical ability. The FIBF model provides a better fit to the empirical data and a cleaner interpretation of mathematical ability than the MIRT and UIRT models.

## 4. Monte Carlo simulation study

In this section, a Monte Carlo simulation is used to illustrate the performance of the four model selection indices and the parameter estimation methods used in the empirical study.

### 4.1. Design

As in the mathematics test in CCEQM 2015–2017, there were six booklets in this simulated test, each including 20 items (12 dichotomous and 8 three-category items). Furthermore, each booklet had items in common with two other booklets; for instance, of the 20 items in “Booklet A,” 10 items were the same as those in “Booklet B,” and 10 items were the same as those in “Booklet C.” Thus, there were *M* = 60 items in total. In addition, as for CCEQM 2015–2017, the 60 items were divided into five dimensions. The sample size of the test takers was *N* = 2, 000, similar to the sample size of students in the empirical study. Each booklet was answered by ≥300 test takers, and each item was included in two booklets. Thus, each item was answered by ≥600 test takers. Overall, the design of this simulation mimics the real situation of CCEQM 2015–2017 as much as possible.

To guarantee that the superior fit of the FIBF model to the empirical data is not due to overfitting, the performance of the four model fit assessment indices (AIC, BIC, SABIC, and HQ) is investigated. In this simulation study, the FIBF, MIRT, and UIRT models were used as the generating models respectively to generate item response data. Let *U*(*l*_1_, *l*_2_) denote the uniform distribution with a range of [*l*_1_, *l*_2_], and *N*(μ, σ^2^) denote the normal distribution with mean μ and variance σ^2^. Let *MVN*(**Λ**, Σ) denote the multivariate normal distribution with mean vector **Λ** and covariance matrix Σ. Based on the results of empirical data analysis, the true values of the item parameters of the three models are selected as follows.

a) *FIBF generating model*

The slope parameters *a*_0*j*_ and *a*_*vj*_ are sampled from


(6) $$\begin{array}{l} a_{0j}\sim U\left(0.5,2.5\right) \end{array}$$

and


(7) $$\begin{array}{l} a_{vj}\sim U\left(0,1.5\right) \end{array}$$

for *v* = 1, ..., 5 and *j* = 1, .., *M*.

The simulated test is a combination of dichotomous and three-category items, and the intercept parameters of the two types of item response models are different. For dichotomous items, the intercept parameter *b*_*j*_ is sampled from


(8) $$\begin{array}{l} b_{j}\sim N\left(0.0,1.0\right), \end{array}$$

while for three-category items, the intercept parameters ***b***_*j*_ = (*b*_1*j*_, *b*_2*j*_) are randomly sampled from


(9) $$\begin{array}{l} b_{1j}\sim U\left(-2.0,0.0\right),b_{2j}\;|b_{1j}\sim U\left(b_{1j},b_{1j}+2.0\right) \end{array}$$

for *j* = 1, ..., *M*.

The latent abilities of the test takers are orthogonal in the FIBF model, so the true values of ***θ***_*i*_ are randomly generated from


(10) $$\begin{array}{l} \theta_{i}\sim MVN(0_{6\times 1},I_{6}) \end{array}$$

for *i* = 1, ..., *N*; here, **0**_6 × 1_ is a 6 × 1 vector in which all elements are 0 and **I**_6_ is the five-dimensional identity matrix.

b) *Between-item MIRT generating model*

The between-item MIRT model can be derived from the FIBF model with the constraint that *a*_0*j*_ = 0 for all items, that is, only the domain-specific slope parameters *a*_*vj*_(*v* = 1, ..., 5) need to be generated. The true value of *a*_*vj*_ is sampled from,


(11) $$\begin{array}{l} a_{vj}\sim U\left(0.5,2.5\right) \end{array}$$

for *v* = 1, ..., 5 and *j* = 1, ..., *M*.

The generation of the true values of the intercept parameters is the same as for the FIBF model, that is, *b*_*j*_ (dichotomous items) and ***b***_*j*_ (three-category items) are sampled from the distributions in Equations (8) and (9).

The latent ability factors of the between-item MIRT model, $\theta_{i}={\left(\theta_{i1},...,\theta_{i5}\right)}^{\prime }$, are correlated, and the true values of ***θ***_*i*_ are sampled from


(12) $$\begin{array}{l} \begin{array}{l} \theta_{i}\sim MVN(0_{5\times 1},{\text{Σ}}_{\theta }), \end{array} \end{array}$$

where Σ**_θ_** is a 5 × 5 covariance matrix, for *i* = 1, ..., *N*. In this simulation, the main diagonal elements of Σ_**θ**_ are fixed to 1, and the remaining elements are covariance parameters that are randomly drawn from a uniform distribution over the range [0.4, 0.8].

c) *UIRT generating model*

The UIRT model can be derived from the FIBF model with the constraint that *a*_*vj*_ = 0 for *v* = 1, ..., 5. There is only one general ability θ_0*i*_ in the UIRT case. The true values of *a*_0*j*_, *b*_*j*_, and *b*_*j*_ are drawn from the distributions in Equations (6), (8), and (9) for *j* = 1, ..., *M*; θ_0*i*_ is randomly drawn from *N*(0, 1) for *i* = 1, ..., *N*.

In this simulation, 20 replications were performed under each simulation condition, and each simulated dataset was fitted by the three models: FIBF, between-item MIRT, and UIRT. All simulations were conducted using the R software, and the four model evaluation indices (AIC, BIC, SABIC, and HQ), as well as the model estimations, were computed based on the “mirt” R package. Note that, if you need the R code, you can contact the authors.

### 4.2. Results

#### 4.2.1. Behavior of model selection indices

The values of AIC, SABIC, HQ, and BIC for the FIBF, MIRT, and UIRT models are reported in Tables 2–**4**.

**Table 2:** Four model selection indices (AIC, SABIC, HQ, and BIC) of the three competing models (FIBF, MIRT, and UIRT) under the condition that the generating model is FIBF.

	**AIC**	**SABIC**	**HQ**	**BIC**		**AIC**	**SABIC**	**HQ**	**BIC**
FIBF	**49737**	**50210**	**50151**	**50858**	FIBF	**49543**	**50016**	**49957**	**50,664**
MIRT	50175	50532	50487	51021	MIRT	49990	50343	50302	50836
UIRT	50652	50986	50944	51443	UIRT	50423	50757	50716	51215
FIBF	**50122**	**50479**	**50434**	**50968**	FIBF	**49773**	**50245**	**50186**	**50894**
MIRT	50652	50986	50944	51443	MIRT	50271	50628	50583	51117
UIRT	50755	51088	51047	51546	UIRT	50755	51088	51047	51546
FIBF	**49750**	**50223**	**50163**	**50871**	FIBF	**49629**	**50102**	**50043**	**50751**
MIRT	50174	50535	50491	51025	MIRT	50110	50467	50423	50957
UIRT	50561	50895	50853	51353	UIRT	50634	50968	50926	51426
FIBF	**49773**	**50246**	**50187**	**50894**	FIBF	**49855**	**50328**	**50269**	**50976**
MIRT	50300	50657	50612	51146	MIRT	50318	50675	50630	51164
UIRT	50749	51083	51041	51540	UIRT	50768	51102	51060	51559
FIBF	**49839**	**50312**	**50253**	**50961**	FIBF	**49920**	**50393**	**50334**	**51041**
MIRT	50222	50579	50534	51068	MIRT	50344	50701	50656	51190
UIRT	50696	51030	50988	51488	UIRT	50765	51099	51057	51557
FIBF	**49863**	**50336**	**50277**	**50984**	FIBF	**49661**	**50134**	**50075**	**50783**
MIRT	50230	50587	50542	51076	MIRT	50181	50538	50493	51027
UIRT	50736	51070	51029	51528	UIRT	50684	51018	50976	51475
FIBF	**49941**	**50414**	**50355**	**51062**	FIBF	**49740**	**50213**	**50154**	**50862**
MIRT	50375	50732	50688	51221	MIRT	50230	50587	50543	51077
UIRT	50836	51170	51128	51627	UIRT	50646	50980	50938	51437
FIBF	**49741**	**50214**	**50155**	**50862**	FIBF	**49947**	**50420**	**50361**	**51068**
MIRT	50172	50529	50485	51019	MIRT	50381	50738	50693	51227
UIRT	50565	50899	50857	51356	UIRT	50834	51168	51127	51626
FIBF	**49516**	**49989**	**49930**	**50638**	FIBF	**49829**	**50302**	**50243**	**50950**
MIRT	50021	50378	50334	50868	MIRT	50335	50692	50647	51181
UIRT	50435	50769	50727	51226	UIRT	50752	51085	51044	51543
FIBF	**49618**	**50091**	**50031**	**50739**	FIBF	**49916**	**50389**	**50329**	**51037**
MIRT	50114	50471	50426	50960	MIRT	50375	50732	50687	51221
UIRT	50525	50859	50818	51317	UIRT	50850	51184	51142	51641

The smallest values of the four model selection indices are bold.

Table 2 presents the results obtained under the condition that the FIBF is the true model. The four model selection indices of the FIBF model are consistently the smallest, which suggests that the FIBF is the best model under this simulation condition. Furthermore, across the 20 replications, the four model evaluation methods consistently suggest that the MIRT model is better than the UIRT model. Because the generating model is FIBF, which is a multidimensional structure, it is correct that the model selection indices support the fit of the MIRT model being better than that of the UIRT model.

Table 3 presents the results obtained under the condition that the between-item MIRT is the true model. All four indices consistently suggest that MIRT, which is the generating model, is the best model. Furthermore, the values of AIC, SABIC, and HQ for the FIBF model are smaller than those for UIRT, whereas the opposite is true for BIC. That is, in comparison with the other model selection methods, the BIC prefers simpler models.

**Table 3:** Four model selection indices (AIC, SABIC, HQ, and BIC) of the three competing models (FIBF, MIRT, and UIRT) under the condition that the generating model is between-item MIRT.

	**AIC**	**SABIC**	**HQ**	**BIC**		**AIC**	**SABIC**	**HQ**	**BIC**
MIRT	**54761**	**55118**	**55073**	**55607**	MIRT	**54904**	**55261**	**55216**	**55750**
FIBF	54868	55341	55282	55989	FIBF	54993	55466	55407	56114
UIRT	55139	55473	55431	55930	UIRT	55378	55712	55670	56109
MIRT	**54676**	**55033**	**54988**	**55522**	MIRT	**54772**	**55129**	**55085**	**55619**
FIBF	54780	55253	55194	55901	FIBF	54936	55409	55350	56057
UIRT	55074	55408	55366	55865	UIRT	55188	55522	55480	55980
MIRT	**54796**	**55153**	**55109**	**55642**	MIRT	**54853**	**55210**	**55165**	**55699**
FIBF	54925	55398	55339	56046	FIBF	54985	55458	55399	56106
UIRT	55085	55419	55377	55876	UIRT	55284	55618	55576	56076
MIRT	**54866**	**55223**	**55178**	**55712**	MIRT	**54638**	**54995**	**54951**	**55485**
FIBF	55007	55480	55421	56129	FIBF	54770	55243	55184	55891
UIRT	55283	55617	55575	56075	UIRT	55084	55417	55376	55875
MIRT	**55003**	**55360**	**55316**	**55849**	MIRT	**54932**	**55289**	**55244**	**55778**
FIBF	55122	55595	55536	56243	FIBF	55043	55516	55456	56164
UIRT	55417	55751	55710	56209	UIRT	55315	55649	55607	56106
MIRT	**54919**	**55276**	**55231**	**55765**	MIRT	**54758**	**55115**	**55071**	**55605**
FIBF	55046	55519	55459	56167	FIBF	54879	55352	55293	56000
UIRT	55286	55619	55578	56077	UIRT	55180	55514	55472	55971
MIRT	**54722**	**55079**	**55034**	**55568**	MIRT	**54637**	**54994**	**54950**	55484
FIBF	54847	55320	55261	55968	FIBF	54739	55212	55152	55860
UIRT	55138	55472	55430	55929	UIRT	54988	55321	55280	55779
MIRT	**54808**	**55165**	**55121**	**55655**	MIRT	**54993**	**55351**	**55306**	**55840**
FIBF	54919	55392	55333	56040	FIBF	55122	55595	55536	56243
UIRT	55079	55412	55371	55870	UIRT	55419	55753	55711	56211
FIBF	**54756**	**55113**	**55069**	**55602**	MIRT	**54721**	**55078**	**55033**	**55567**
MIRT	54869	55342	55283	55990	FIBF	54858	55331	55272	55979
UIRT	55130	55464	55422	55921	UIRT	55156	55490	55448	55947
MIRT	**54824**	**55181**	**55137**	**55671**	MIRT	**54796**	**55153**	**55108**	**55642**
FIBF	54935	55408	55348	56056	FIBF	54906	55379	55320	56027
UIRT	55222	55555	55514	56013	UIRT	55178	55512	55470	55970

The smallest values of the four model selection indices are bold.

Table 4 presents the results obtained under the condition that the UIRT is the true model. As for the above two simulation conditions, the four model evaluation indices consistently indicate that the generating model is the best model. Except for AIC, the evaluation indices indicate that MIRT is better than FIBF. We believe that MIRT and FIBF should be very close in fitting the unidimensional test data, but as the MIRT model is simpler, it is preferred by the SABIC, HQ, and BIC indices.

**Table 4:** Four model selection indices (AIC, SABIC, HQ, and BIC) of the three competing models (FIBF, MIRT, and UIRT) under the condition that the generating model is UIRT.

	**AIC**	**SABIC**	**HQ**	**BIC**		**AIC**	**SABIC**	**HQ**	**BIC**
UIRT	**45512**	**45845**	**45804**	**46303**	UIRT	**45457**	**45789**	**45747**	**46243**
FIBF	45548	46021	45962	46669	FIBF	45500	45971	45912	46615
MIRT	45633	45990	45945	46479	MIRT	45553	45908	45863	46394
UIRT	**45106**	**45440**	**45398**	**45897**	UIRT	**45406**	**45738**	**45696**	**46192**
FIBF	45150	45623	45564	46271	FIBF	45425	45896	45837	46541
MIRT	45201	45535	45487	46017	MIRT	45507	45862	45817	46348
UIRT	**45397**	**45728**	**45687**	**46182**	UIRT	**45430**	**45764**	**45722**	**46221**
FIBF	45440	45911	45852	46556	FIBF	45478	45951	45892	46599
MIRT	45493	45848	45804	46334	MIRT	45539	45896	45851	46385
UIRT	**45799**	**46133**	**46091**	**46590**	UIRT	**45430**	**45763**	**45722**	**46221**
FIBF	45856	46329	46270	46977	FIBF	45468	45941	45881	46589
MIRT	45915	46272	46228	46761	MIRT	45501	45858	45813	46347
UIRT	**45574**	**45906**	**45864**	**46360**	UIRT	**45511**	**45843**	**45801**	**46297**
FIBF	45622	46093	46034	46738	FIBF	45561	46031	45973	46676
MIRT	45672	46027	45983	46513	MIRT	45613	45968	45923	46454
UIRT	**45030**	**45364**	**45322**	**45821**	UIRT	**45536**	**45870**	**45828**	**46327**
FIBF	45084	45557	45498	46205	FIBF	45572	46045	45986	46693
MIRT	45130	45487	45442	45976	MIRT	45653	46010	45965	46499
UIRT	**45632**	**45989**	**45944**	**46478**	UIRT	**45327**	**45658**	**45617**	**46113**
FIBF	45737	46207	46149	46852	FIBF	45374	45844	45785	46489
MIRT	45737	46068	46027	46523	MIRT	45437	45792	45748	46278
UIRT	**45545**	**45879**	**45837**	**46337**	UIRT	**45710**	**46042**	**46001**	**46496**
FIBF	45592	46065	46006	46713	FIBF	45757	46228	46169	46873
MIRT	45640	45996	45952	46486	MIRT	45805	46159	46115	46646
UIRT	**45200**	**45532**	**45490**	**45986**	UIRT	**45353**	**45684**	**45643**	**46138**
FIBF	45237	45707	45648	46352	FIBF	45385	45855	45797	46500
MIRT	45317	45671	45627	46157	MIRT	45469	45824	45780	46310
UIRT	**44973**	**45307**	**45265**	**45764**	UIRT	**45261**	**45595**	**45553**	**46052**
FIBF	45004	45477	45418	46125	FIBF	45288	45761	45701	46409
MIRT	45084	45441	45396	45930	MIRT	45364	45721	45676	46210

The smallest values of the four model selection indices are bold.

Summarizing these results, the four model selection indices (AIC, BIC, SABIC, and HQ) provide excellent accuracy in assessing the three models used in the empirical study. First, they successfully identified the model used to generate the item response data across all simulation conditions. Second, when the FIBF was not the generating model, some model selection indices such as BIC did not support the FIBF model being better than the simpler models. This indicates that overfitting of the bifactor model does not occur for the FIBF model under the incomplete block design.

#### 4.2.2. Recovery of item parameters

The recovery of the three models (FIBF, MIRT, and UIRT) using the “mirt” package was checked in this simulation. The estimation accuracy was assessed by computing the average root mean squire error (ARMSE) of each parameter over all items and the average of the correlation (ACor) between the estimates and the true parameter across the 20 replications.

These metrics were calculated as


$$\begin{array}{l} {\text{ARMSE}}_{\hat{\delta }}=\frac{1}{M}\overset{M}{\underset{j=1}{\sum }}\sqrt{20^{-1}\overset{20}{\underset{g=1}{\sum }}{\left({\hat{\delta }}_{jg}-\delta_{j}\right)}^{2}} \end{array}$$

and


$$\begin{array}{l} {\text{ ACor}}_{\hat{\delta }}\;\\ =\frac{1}{20}\sum_{g=1}^{20}\frac{\sum_{j=1}^{M}{\hat{\delta }}_{jg}\delta_{j}-\frac{1}{M}\sum_{j}^{M}{\hat{\delta }}_{jg}\sum_{j}^{M}\delta_{j}}{\sqrt{\left(\sum_{j=1}^{M}{\hat{\delta }}_{jg}^{2}-\frac{1}{M}{\left(\sum_{j=1}^{M}{\hat{\delta }}_{jg}\right)}^{2})(\sum_{j=1}^{M}\delta_{j}^{2}-\frac{1}{M}{\left(\sum_{j=1}^{M}\delta_{j}\right)}^{2}\right)}}, \end{array}$$

where δ_*j*_ denotes any one of the parameters of item *j* and ${\hat{\delta }}_{jg}$ denotes the corresponding estimate obtained with the *g*-th simulated data.

The obtained results are presented in Table 5. Use of the “mirt” package allows the item parameters of the three models to be recovered satisfactorily under simulated conditions that are similar to the math tests in CCEQM 2015–2017. First, for all three models, the ARMSE values of the slope parameters do not exceed 0.3, and the corresponding correlations range from 0.94 to 0.99. These results indicate that the slope parameters were well-recovered. Second, the ARMSEs of the intercept parameters of the three models are 0.15, and the values of ACor are >0.98. The recovery for the intercept parameters is excellent, and is slightly better than that for the slope parameters. Furthermore, the estimation accuracy of FIBF is slightly poorer than that of MIRT and UIRT. As stated above, FIBF is the most complex of the three models, so it is normal that the accuracy of its parameter estimation is slightly poorer. Finally, each item was answered by no more than 700 test takers in this simulation. It is likely that the recovery accuracy of the three models will improve as the sample size increases.

**Table 5:** ARMSE (ACor) values for the estimation of the item slope and intercept parameters in the three models: FIBF, between-item MIRT, and UIRT.

	**General-factor slope**	**Specific-factor slope**					**Intercept**
	***a*_0_**	***a*_1_**	***a*_2_**	***a*_3_**	***a*_4_**	***a*_5_**	***b***
FIBF	0.19 (0.95)	0.27 (0.95)	0.29 (0.94)	0.27 (0.97)	0.29 (0.95)	0.28 (0.94)	0.15 (0.98)
MIRT	–	0.24 (0.98)	0.28 (0.97)	0.32 (0.99)	0.25 (0.99)	0.26 (0.99)	0.15 (0.99)
UIRT	0.19 (0.97)	–	–	–	–	–	0.15 (0.99)

## 5. Conclusion and further issues

The main contribution of this study is to propose a bifactor structure for modeling mathematical ability. On this basis, a mixed FIBF model has been developed for the measurement of mathematical ability in CCEQM 2015–2017. Within the discipline of core literacy theory, mathematical ability is defined as a construct that consists of several domains. These domains differ in their specific concept, but they have common elements, and are thus highly correlated because they all belong to the broader concept of mathematical ability. Bifactor models are a powerful approach for describing such constructs, in which the common element of the five domains is represented by a general factor that is considered as general mathematical ability, and the uniqueness of each domain is represented by a group or domain-specific factor. From the view of bifactor theory, this study has proposed a mixed FIBF model that is a bifactor extension of the mixed IRT model for the measurement of mathematical ability in CCEQM 2015–2017. Furthermore, an important advantage of FIBF analysis is its wide practical adaptability. FIBF models can be applied to various test situations, and numerous related computing tools have been developed. These provide strong support for the application of FIBF to actual large-scale tests. Taken together, not only is the bifactor structure a reasonable approach for representing mathematical ability, but FIBF models are also highly feasible in practice.

The second contribution of this study comes from the empirical study conducted using data from CCEQM 2018 to verify the performance of the FIBF model. The results for the four model selection indices (AIC, BIC, SABIC, and HQ) consistently showed that the fit of the FIBF model is better than that of the UIRT and MIRT models, and the ability scores from the FIBF model had a more reasonable interpretation. The advantages of the FIBF model are fully verified by this empirical study. One important problem with the application of bifactor models is their ease of overfitting. To ensure that no overfitting occurred in the empirical study, a Monte Carlo simulation was constructed to investigate the performance of the four model selection indices under a test design similar to that of CCEQM 2015–2017. The results indicate that the four indices consistently select the generating or true model as the best model across all simulation conditions, and when the FIBF is not the true model, it is not supported. Therefore, the model selection results in the empirical study provide strong evidence that, for fitting the math test data, the FIBF model is superior to the MIRT and UIRT approaches. Furthermore, the simulation results demonstrate that the estimations of the FIBF model have high recovery accuracy under the incomplete test design with mixed item types. Overall, the simulation results show that the existing methods and technologies can support the application of the FIBF model in large-scale testing projects.

There are several considerations for the current study that warrant mention. (1) The estimations of domain-specific factors cannot be directly regarded as scale scores of domain-specific abilities, because they only represent the uniqueness of domain-specific abilities. The scale score of each domain should be a combination of the general and domain-specific factors. Therefore, computing the scale score of domain-specific ability is an important issue that requires further study. (2) Heterogeneity is an important issue in large-scale assessments, such as measurement invariance, heterogeneity of residual variance, and whether the distribution of latent ability is multimodal or skewed. These factors inevitably result in serious unfairness in testing. Thus, to ensure fairness in testing, the development and application of a heterogeneity FIBF model should be further studied. (3) In this study, the bifactor structure of mathematical ability was verified by only one empirical study, and so the obtained conclusion has certain limitations. The bifactor assumption of mathematical ability should be discussed based on more empirical data from large-scale assessment projects. (4) The application of bifactor theory to a measurement model for other disciplines is also worthy of further study.

## Data availability statement

The original contributions presented in the study are included in the article, further inquires can be directed to the corresponding authors.

## Author contributions

XM contributed to implementing the studies and writing the initial draft. TY and TX contributed to providing the data, key technical support, and manuscript revision. NS contributed to conceptualizing ideas, key technical support and providing a few suggestions on the focus, and direction of the research. All authors contributed to the article and approved the submitted version.

## References

[B1] Acton G. S. Schroeder D. H. (2001). Sensory discrimination as related to general intelligence. Intelligence 29, 263–271. 10.1016/S0160-2896(01)00066-6

[B2] Akaike H. (1987). Factor analysis and the AIC. Psychometrika 52, 317–332. 10.1007/BF02294359

[B3] Arens A. K. Jansen M. Preckel F. Schmidt I. Brunner M. (2021). The structure of academic self-concept: a methodological review and empirical illustration of central models. Rev. Educ. Res. 91, 34–72. 10.3102/0034654320972186

[B4] Beaujean A. A. Parkin J. Parker S. (2014). Comparing Cattell-Horn-Carroll factor models: differences between bifactor and higher order factor models in predicting language achievement. Psychol. Assess. 26, 789–805. 10.1037/a0036745 24840178

[B5] Bonifay W. Cai L. (2017). On the complexity of item response theory models. Multivar. Behav. Res. 52, 465–484. 10.1080/00273171.2017.1309262 28426237

[B6] Bornovalova M. A. Choate A. M. Fatimah H. Petersen K. J. Wiernik B. M. (2020). Appropriate use of bifactor analysis in psychopathology research: appreciating benefits and limitations. Biol. Psychiatry 88, 18–27. 10.1016/j.biopsych.2020.01.013 32199605 PMC10586518

[B7] Cai L. (2010). High-dimensional exploratory item factor analysis by a Metropolis-Hastings Robbins-Monro algorithm. Psychometrika 75, 33–57. 10.1007/s11336-009-9136-x33528784

[B8] Cai L. Yang J. S. Hansen M. (2011). Generalized full-information item bifactor analysis. Psychol. Methods 16, 221–248. 10.1037/a0023350 21534682 PMC3150629

[B9] Caiado B. Canavarro M. C. Moreira H. (2022). The bifactor structure of the emotion expression scale for children in a sample of school-aged Portuguese children. Assessment 1–15. 10.1177/10731911221082038. [Epub ahead of print]. 35272501 PMC10152220

[B10] Chen F. F. West S. G. Sousa K. H. (2006). A comparison of bifactor and second-order models of quality of life. Multivar. Behav. Res. 41, 189–225. 10.1207/s15327906mbr4102_5 26782910

[B11] Cucina J. Byle K. (2017). The bifactor model fits better than the higher-order model in more than 90% of comparisons for mental abilities test batteries. J. Intell. 5, 1–27. 10.3390/jintelligence5030027 31162418 PMC6526460

[B12] DeMars C. E. (2006). Application of the bi-factor multidimensional item response theory model to testlet-based tests. J. Educ. Measure. 43, 145–168. 10.1111/j.1745-3984.2006.00010.x

[B13] Foorman B. R. Koon S. Petscher Y. Mitchell A. Truckenmiller A. (2015). Examining general and specific factors in the dimensionality of oral language and reading in 4th-10th grades. J. Educ. Psychol. 107:884. 10.1037/edu0000026 26346839 PMC4557887

[B14] Gault U. (1954). Factorial patterns of the Wechsler intelligence scales. Austr. J. Psychol. 6, 85–89. 10.1080/00049535408256079

[B15] Gibbons R. D. Bock R. D. Hedeker D. Weiss D. J. Segawa E. Bhaumik D. K. . (2007). Full-information item bifactor analysis of graded response data. Appl. Psychol. Measure. 31, 4–19. 10.1177/0146621606289485

[B16] Gibbons R. D. Hedeker D. R. (1992). Full-information item bi-factor analysis. Psychometrika 57, 423–436. 10.1007/BF02295430

[B17] Gomez R. McLaren S. (2015). The center for epidemiologic studies depression scale: support for a bifactor model with a dominant general factor and a specific factor for positive affect. Assessment 22, 351–360. 10.1177/1073191114545357 25085880

[B18] Greene A. L. Eaton N. R. Li K. Forbes M. K. Krueger R. F. Markon K. E. . (2019). Are fit indices used to test psychopathology structure biased? A simulation study. J. Abnorm. Psychol. 128:740. 10.1037/abn0000434 31318246

[B19] Hannan E. J. Quinn B. G. (1979). The determination of the order of an autoregression. J. R. Stat. Soc. Ser. B Methodol. 41, 190–195. 10.1111/j.2517-6161.1979.tb01072.x

[B20] Heinrich M. Zagorscak P. Eid M. Knaevelsrud C. (2020). Giving G a meaning: an application of the bifactor-(S-1) approach to realize a more symptom-oriented modeling of the Beck depression inventory-II. Assessment 27, 1429–1447. 10.1177/1073191118803738 30293444

[B21] Holzinger K. J. Swineford F. (1937). The bi-factor method. Psychometrika 2, 41–54. 10.1007/BF02287965

[B22] Immekus J. C. Imbrie P. (2008). Dimensionality assessment using the full-information item bifactor analysis for graded response data: an illustration with the state metacognitive inventory. Educ. Psychol. Measure. 68, 695–709. 10.1177/0013164407313366

[B23] Jiang Y. Zhang J. Xin T. (2019). Toward education quality improvement in China: a brief overview of the national assessment of education quality. J. Educ. Behav. Stat. 44, 733–751. 10.3102/1076998618809677

[B24] Jorge-Botana G. Olmos R. Luzón J. M. (2019). Could LSA become a “bifactor” model? Towards a model with general and group factors. Expert Syst. Appl. 131, 71–80. 10.1016/j.eswa.2019.04.055

[B25] Kim H. Eaton N. R. (2015). The hierarchical structure of common mental disorders: connecting multiple levels of comorbidity, bifactor models, and predictive validity. J. Abnorm. Psychol. 124:1064. 10.1037/abn0000113 26595482

[B26] Kim K. Y. Cho U. H. (2020). Approximating bifactor IRT true-score equating with a projective item response model. Appl. Psychol. Measure. 44, 215–218. 10.1177/0146621619885903 32341608 PMC7174803

[B27] Leue A. Beauducel A. (2011). The PANAS structure revisited: on the validity of a bifactor model in community and forensic samples. Psychol. Assess. 23:215. 10.1037/a0021400 21280952

[B28] Li Y. Lissitz R. W. (2012). Exploring the full-information bifactor model in vertical scaling with construct shift. Appl. Psychol. Measure. 36, 3–20. 10.1177/0146621611432864

[B29] Liu Y. Thissen D. (2012). Identifying local dependence with a score test statistic based on the bifactor logistic model. Appl. Psychol. Measure. 36, 670–688. 10.1177/0146621612458174

[B30] Martel M. M. Roberts B. Gremillion M. Von Eye A. Nigg J. T. (2011). External validation of bifactor model of ADHD: explaining heterogeneity in psychiatric comorbidity, cognitive control, and personality trait profiles within DSM-IV ADHD. J. Abnorm. Child Psychol. 39, 1111–1123. 10.1007/s10802-011-9538-y 21735050 PMC3199328

[B31] McAbee S. T. Oswald F. L. Connelly B. S. (2014). Bifactor models of personality and college student performance: a broad versus narrow view. Eur. J. Pers. 28, 604–619. 10.1002/per.1975

[B32] McFarland D. J. (2013). Modeling individual subtests of the WAIS IV with multiple latent factors. PLoS ONE 8:e74980. 10.1371/journal.pone.0074980 24058643 PMC3772883

[B33] McFarland D. J. (2016). Modeling general and specific abilities: evaluation of bifactor models for the WJ-III. Assessment 23, 698–706. 10.1177/1073191115595070 26187901

[B34] Monteiro F. Fonseca A. Pereira M. Canavarro M. C. (2021). Measuring positive mental health in the postpartum period: the bifactor structure of the mental health continuum-short form in Portuguese women. Assessment 28, 1434–1444. 10.1177/1073191120910247 32167379

[B35] Morgan G. B. Hodge K. J. Wells K. E. Watkins M. W. (2015). Are fit indices biased in favor of bi-factor models in cognitive ability research?: a comparison of fit in correlated factors, higher-order, and bi-factor models *via* Monte Carlo simulations. J. Intell. 3, 2–20. 10.3390/jintelligence3010002

[B36] Moshagen M. Hilbig B. E. Zettler I. (2018). The dark core of personality. Psychol. Rev. 125:656. 10.1037/rev0000111 29999338

[B37] Murray A. L. Johnson W. (2013). The limitations of model fit in comparing the bi-factor versus higher-order models of human cognitive ability structure. Intelligence 41, 407–422. 10.1016/j.intell.2013.06.004

[B38] OECD (2014). PISA 2012 Technical Report. OECD.

[B39] Olatunji B. O. Ebesutani C. Abramowitz J. S. (2017). Examination of a bifactor model of obsessive-compulsive symptom dimensions. Assessment 24, 45–59. 10.1177/1073191115601207 26310961

[B40] Reise S. P. (2012). The rediscovery of bifactor measurement models. Multivar. Behav. Res. 47, 667–696. 10.1080/00273171.2012.715555 24049214 PMC3773879

[B41] Reise S. P. Morizot J. Hays R. D. (2007). The role of the bifactor model in resolving dimensionality issues in health outcomes measures. Qual. Life Res. 16, 19–31. 10.1007/s11136-007-9183-7 17479357

[B42] Rodriguez A. Reise S. P. Haviland M. G. (2016). Evaluating bifactor models: Calculating and interpreting statistical indices. Psychol. Methods 21:137. 10.1037/met0000045 26523435

[B43] Rushton J. P. Irwing P. (2009a). A general factor of personality (GFP) from the multidimensional personality questionnaire. Pers. Individ. Differ. 47, 571–576. 10.1016/j.paid.2009.05.011

[B44] Rushton J. P. Irwing P. (2009b). A general factor of personality in 16 sets of the Big Five, the Guilford-Zimmerman Temperament Survey, the California Psychological Inventory, and the Temperament and Character Inventory. Pers. Individ. Differ. 47, 558–564. 10.1016/j.paid.2009.05.009

[B45] Schwarz G. (1978). Estimating the dimension of a model. Ann. Stat. 6, 461–464. 10.1214/aos/1176344136

[B46] Sclove S. L. (1987). Application of model-selection criteria to some problems in multivariate analysis. Psychometrika 52, 333–343. 10.1007/BF02294360

[B47] Sellbom M. Tellegen A. (2019). Factor analysis in psychological assessment research: common pitfalls and recommendations. Psychol. Assess. 31:1428. 10.1037/pas0000623 31120298

[B48] Shevlin M. McElroy E. Bentall R. P. Reininghaus U. Murphy J. (2016). The psychosis continuum: testing a bifactor model of psychosis in a general population sample. Schizophr. Bull. 43, 133–141. 10.1093/schbul/sbw067 27220965 PMC5216850

[B49] Simms L. J. Grös D. F. Watson D. O'Hara M. W. (2008). Parsing the general and specific components of depression and anxiety with bifactor modeling. Depress. Anxiety 25, E34–E46. 10.1002/da.20432 18027844

[B50] Snyder H. R. Hankin B. L. Sandman C. A. Head K. Davis E. P. (2017). Distinct patterns of reduced prefrontal and limbic gray matter volume in childhood general and internalizing psychopathology. Clin. Psychol. Sci. 5, 1001–1013. 10.1177/2167702617714563 29399423 PMC5794221

[B51] Spearman C. (1904). General ability, objectively determined and measured. Am. J. Psychol. 15:201. 10.2307/1412107

[B52] The National Assessment Center for Education Quality (2018). The China Compulsory Education Quality Oversight Report [in Chinese]. Available online at: http://www.moe.gov.cn/jyb_xwfb/moe_1946/fj_2018/201807/P020180724685827455405.pdf

[B53] Valerius S. Sparfeldt J. R. (2014). Consistent g- as well as consistent verbal-, numerical- and figural-factors in nested factor models? Confirmatory factor analyses using three test batteries. Intelligence 44, 120–133. 10.1016/j.intell.2014.04.003

[B54] Waldman I. D. Poore H. E. Luningham J. M. Yang J. (2020). Testing structural models of psychopathology at the genomic level. World Psychiatry 19, 350–359. 10.1002/wps.20772 32931100 PMC7491626

[B55] Watkins M. W. (2010). Structure of the Wechsler intelligence scale for children-Fourth edition among a national sample of referred students. Psychol. Assess. 22:782. 10.1037/a0020043 21133545

[B56] Watkins M. W. Beaujean A. A. (2014). Bifactor structure of the Wechsler preschool and primary scale of intelligence-fourth edition. School Psychol. Q. 29:52. 10.1037/spq0000038 24188289

[B57] Yin D. (2021). Education quality assessment in China: what we learned from official reports released in 2018 and 2019. ECNU Rev. Educ. 4, 396–409. 10.1177/2096531120944522

[B58] Zhan P. Yu Z. Li F. Wang L. (2019). Using a multi-order cognitive diagnosis model to assess scientific literacy. Acta Psychol. Sin. 51:734. 10.3724/SP.J.1041.2019.00734

